# Comprehensive analysis to identify GNG7 as a prognostic biomarker in lung adenocarcinoma correlating with immune infiltrates

**DOI:** 10.3389/fgene.2022.984575

**Published:** 2022-09-09

**Authors:** Qin Wei, Tianshu Miao, Pengju Zhang, Baodong Jiang, Hua Yan

**Affiliations:** ^1^ Department of Biochemistry and Molecular Biology, Shandong University School of Basic Medical Sciences, Jinan, China; ^2^ Department of Radiology, Qilu Hospital of Shandong University, Jinan, China; ^3^ Department of Gastroenterology, Affiliated Hospital of Shandong University of Traditional Chinese Medicine, Jinan, China

**Keywords:** immune microenvironment 1, GNG7 2, prognosis 3, lung adenocarcinoma 4, bioinformatics analysis 5

## Abstract

**Background:** G Protein Subunit Gamma 7 (GNG7), an important regulator of cell proliferation and cell apoptosis, has been reported to be downregulated in a variety of tumors including lung adenocarcinoma (LUAD). However, the correlation between low expression of GNG7 and prognosis of LUAD as well as the immune infiltrates of LUAD remains unclear.

**Methods:** The samples were obtained from The Cancer Genome Atlas (TCGA) and Gene Expression Omnibus (GEO). R software was performed for statistical analysis. GNG7 expression and its prognostic value in LUAD were assessed through statistically analyzing the data from different databases. A nomogram was constructed to predict the impact of GNG7 on prognosis. Gene set enrichment analysis (GSEA) and single-sample gene set enrichment analyses GSEA (ssGSEA) were employed to determine the potential signal pathways and evaluated the immune cell infiltration regulated by GNG7. The prognostic significance of GNG7 expression associated with immune cell infiltration was investigated using the Tumor Immune Estimation Resource 2.0 (TIMER2.0) and the Kaplan-Meier plotter database. The UALCAN, cBio Cancer Genomics Portal (cBioPortal) and MethSurv database were used to analyze the correlation between the methylation of GNG7 and its mRNA expression as well as prognostic significance.

**Results:** GNG7 was demonstrated to be down-regulated in LUAD and its low expression was associated with poor prognosis. A clinical reliable prognostic-predictive model was constructed. Pathway enrichment showed that GNG7 was highly related to the B cell receptor signaling pathway. Further analysis showed that GNG7 was positively associated with B cell infiltration and low levels of B cell infiltration tended to associate with worse prognosis in patients with low GNG7 expression. Moreover, methylation analysis suggested hypermethylation may contribute to the low expression of GNG7 in LUAD.

**Conclusion:** Decreased expression of GNG7 at least partly caused by hypermethylation of the GNG7 promoter is closely associated with poor prognosis and tumor immune cell infiltration (especially B cells) in LUAD. These results suggest that GNG7 may be a promising prognostic biomarker and a potential immunotherapeutic target for LUAD, which provides new insights into immunotherapy for LUAD.

## Introduction

Lung cancer is the most common reason for global cancer-related mortality, of which lung adenocarcinoma (LUAD) is the most common histological subtype (Travis, 2011; Sung et al., 2021). In recent years, although molecular targeted therapies and immunotherapy have significantly improved the prognosis of a small proportion of LUAD patients, the above treatments are ineffective in many patients due to the high heterogeneity and complexity of LUAD (Molina et al., 2008; Saito et al., 2018; Wu and Shih, 2018). The prognosis for many patients, especially those with advanced LUAD, remains poor, with a 5-year survival rate of less than 18% (Singh et al., 2020). Therefore, an in-depth pathogenetic exploration and search for other effective diagnostic and therapeutic approaches as well prognostic markers are essential to improve the prognosis of patients with LUAD.

Accumulating evidence suggests that the immune cells within the tumor microenvironment (TME) play essential roles in tumorigenesis (Hinshaw and Shevde, 2019). Such tumor associated immune cells may exert pro-tumor or anti-tumor function in the initiation and development of tumors (Taube et al., 2018). Studies have shown that immune cell infiltration is an important factor influencing the efficacy of immunotherapy (Martinez and Moon, 2019; Petitprez et al., 2020; Bagchi et al., 2021). In addition, TME is also closely related to the prognosis of patients (Qi et al., 2020; Wu et al., 2020). Therefore, it is crucial to investigate the regulators of immune cell infiltration in the TME to improve the effectiveness of immunotherapy and improve patient prognosis.

G Protein Subunit Gamma 7 (GNG7), a subunit of heterotrimeric G protein, is strongly enriched in the striatum and plays a vital role in the A2A adenosine or D1 dopamine receptor-induced neuro-protective response (Schwindinger et al., 2012). Multiple studies have shown that GNG7 expression is decreased in many cancers, including pancreatic cancer, gastrointestinal tract cancer and renal carcinoma (Shibata et al., 1998; Shibata et al., 1999; Ohta et al., 2008). GNG7 overexpression was shown to inhibit cell growth and tumorigenicity of esophageal carcinoma cells (Hartmann et al., 2012). Also, GNG7 was confirmed as an essential autophagy-inducing agent and participated in inhibiting tumor progression through mTOR pathway (Xu et al., 2019). Recently, GNG7 was reported to be lowly expressed in LUAD and promoted the progression of LUAD through Hedgehog signaling (Liu et al., 2016). These findings indicated that GNG7 may be a potential tumor suppressor implicated in the carcinogenesis and tumor progression. However, the detailed roles and mechanisms of GNG7 especially the effects of GNG7 on immune infiltration in LUAD are largely unknown.

In this study, we aimed to evaluate the clinical significance of GNG7 in LUAD and the possible mechanisms underlying its function through comprehensive bioinformatics analysis. Our results showed that low expression of GNG7 positively correlated with the progression of LUAD, and GNG7 may be an important potential prognostic biomarker for LUAD. We also constructed a reliable clinical prediction model. In addition, we revealed for the first time the underlying mechanisms of GNG7 dysregulation and the correlation between GNG7 expression and immune infiltration in LUAD, which implies that GNG7 could be a potential target for clinical antitumor immunotherapy.

## Materials and methods

### Data sources and pretreatment

The RNA-seq data of 513 LUAD samples and 59 normal samples were downloaded from The Cancer Genome Atlas (TCGA) database (https://portal.gdc.cancer.gov/). The downloaded data format was level 3 HTSeq-fragments per kilobase per million (FPKM) and then was converted to transcripts per million (TPM) format for subsequent analysis. TCGA supplemented prognostic data were obtained from a Cell article (Liu et al., 2018). In addition, three sets of microarray data of LUAD tissues (accession numbers: GSE32665, GSE32863, and GSE43458) were downloaded from the GEO database. The data used in this study were obtained from both the TCGA database and the GEO database, which ensured that all written informed consent was obtained prior to data collection.

### Key gene screening

The R package “DESeq2” was used to identify the differentially expressed genes (DEGs) between LUAD tissues and normal tissues (Love et al., 2014). Adjusted *p*-value <0.05 and | Log2fold change| >1 were set as cut-off criteria. The Survminer R package and the survivor R package were used to screen for genes with better prognostic value. The cut-off threshold was hazard ratio (HR) < 1, and the Cox *p*-value <0.005. The prognostic indicators included overall survival (OS), disease-specific survival (DSS) and progression-free survival (PFS). The Venn diagram was used to represent the intersection set of DEGs obtained from the four sets.

### G protein subunit gamma 7 differential expression analysis in lung adenocarcinoma tissues

Pre-processed TCGA-LUAD data were used for differential expression analysis of GNG7 in LUAD tumor tissues and normal tissues. Three GEO expression profile datasets, GSE32665, GSE32863, and GSE43458, were used to compare the expression of GNG7 between LUAD tissues and normal tissues. Differential protein levels of GNG7 between LUAD tissues and normal tissues were analyzed by UALCAN (http://ualcan.path.uab.edu/) (Chandrashekar et al., 2022). A receiver operating characteristic (ROC) curve was used to evaluate the diagnostic significance of GNG7 using the plotROC R package (Version 1.17.0.1) (Robin et al., 2011).

### Clinical statistical analysis on prognosis, model construction and evaluation

The correlation between GNG7 expression and survival in LUAD patients was analyzed by the PrognoScan database (http://www.prognoscan.org/) (Mizuno et al., 2009). Univariate Cox regression analysis, multivariate Cox regression analysis, logistic analysis and Kaplan-Meier (K-M) analysis were employed for prognosis analysis. The independent prognostic factors obtained from multivariate Cox regression analysis were employed to establish nomograms to predict survival probability for 1-, 2-, and 3-year. The calibration curves and nomograms were analyzed and plotted *via* the rms (version 6.2-0) and survival (version 3.2-10) package of R software. The calibration curves were graphically assessed by mapping the nomogram-predicted probabilities against the observed rates, and the 45-degree line represented the best predictive values. A concordance index (C-index) was used to determine the discrimination of the nomogram. According to the median risk score, patients were divided into a high-risk score group and a low-risk score group. The survival difference between the two groups was assessed by K-M survival curves. The model was compared with the two-by-two model consisting of independent prognostic factors screened from multivariate Cox regression, and ROC curves made by the timeROC R package (version 0.4) were used to assess the accuracy of the model predictions. The risk curve was used to demonstrate the application of the model in predicting clinical outcomes.

### Gene set enrichment analysis

DESeq2 package (Version 1.26.0) was employed to identify the DEGs between GNG7-high and GNG7-low expression patients from TCGA datasets. The cut-off threshold was |log fold change (FC)|>1 and adjusted *p*-value <0.05. All the DEGs were presented in the volcano plots, and the correlation of some representative DEGs with GNG7 was presented in heatmaps. Gene Set Enrichment Analysis (GSEA) is a computational method for determining whether a defined set of genes shows statistically significant differences between two states. In the study, GSEA was performed by using the clusterProfiler R package (version 3.14.3) with c2 (c2. all.v7.0. entrez.gmt) from the Molecular Signatures Database (MSigDB) (Subramanian et al., 2005; Yu et al., 2012). Each analysis procedure was repeated 1000 times. The function or pathway termed with adjusted *p*-value <0.05 and false discovery rate (FDR) < 0.25 was considered statistically significant enrichment.

### Tumor immune infiltration analysis, protein-protein interaction network analyses and the screening of hub genes

ESTIMATE algorithm was used to calculate the immune scores using the “estimate” R package (Yoshihara et al., 2013). Single-sample gene set enrichment analysis (ssGSEA) algorithm was used to assess the relative enrichment of the tumor tissue-infiltrating immune cells in LUAD (Hänzelmann et al., 2013). Based on an immune dataset for the 24 types of immunocytes, the relative enrichment score of every immunocyte was quantified from the gene expression profiles of each tumor sample (Bindea et al., 2013). In addition, we analyzed the differences in the enrichment of these 24 immune cells between the high and low GNG7 expression groups using ssGSEA. The Stat R package (Version 3.6.3) was employed to search for the genes related to GNG7 in LUAD. The correlation results were analyzed by the Spearman coefficient and the cut-off thresholds were |R| >0.4 and *p*-value <0.05. The list of immune-related genes was obtained from the ImmPort database. Genes associated with GNG7 were intersected with IRGs to obtain IRGs associated with GNG7. To further understand the interactions between IRGs associated with GNG7, we constructed a Protein-Protein Interaction (PPI) using the Search Tool for the Retrieval of Interacting Genes (STRING) (https://string-db.org/). An interaction with a combined score >0.4 was considered statistically significant. Cytoscape (Version 3.7.2) was used to visualize the network, while the cytoHubba plugin was used to rank genes within this network based upon their degree centrality values. Hub genes were considered to be those with the top 10 highest degree values. ClusterProfiler R package was employed to perform Gene Ontology (GO) function enrichment analyses. Furthermore, We used the GEPIA2 database (http://gepia2.cancer-pku.cn/#index) to analyze the correlation between GNG7 and B cell infiltration in LUAD tissues and normal tissues (Tang et al., 2019). Tumor Immune Estimation Resource 2.0 (TIMER2.0) database (http://timer.cistrome.org/) and the Kaplan-Meier plotter database (http://kmplot.com/analysis/) were used for the prognostic analysis of LUAD patients with different GNG7 expression and B cell infiltration (Li T. et al., 2020; Lánczky and Győrffy, 2021). We also used the TIMER database to analyze the correlation between GNG7 and immune cell markers in LUAD (Li et al., 2017).

### DNA methylation analysis

To explore the possible mechanism of decreased expression of GNG7 in LUAD, we performed a differential methylation analysis of GNG7 between the normal and LUAD tissues using the UALCAN database. The cBio Cancer Genomics Portal (cBioPortal) (https://www.cbioportal.org), developed based on the TCGA database, was used to perform a correlation analysis between GNG7 mRNA expression and its methylation levels (Cerami et al., 2012; Gao et al., 2013). DNA methylation of GNG7 at CpG sites and the prognostic value of these CpG sites in LUAD were analyzed by MethSurv (https://biit.cs.ut.ee/methsurv/) (Modhukur et al., 2018).

### Statistical analysis

All statistical analyses were conducted using R (Version 3.6.3). A part of the figures was plotted using the ggplot2 R package (Version 3.3.3). Dunn’s test, Kruskal–Wallis test, and logistic regression were used to analyze the clinicopathological features of GNG7 in LUAD. Kaplan-Meier survival analysis, univariate and multivariate Cox regression analysis were performed for prognostic analysis. In all analyses, the *p*-value<0.05 was considered statistically significant. The specific datasets, R packages, software and databases used in each part of this study are detailed in Supplementary Table S4.

## Results

### G protein subunit gamma 7 is found to be one of the key regulators of lung adenocarcinoma tightly related to the prognosis through large-scale screening

**FIGURE 1:**
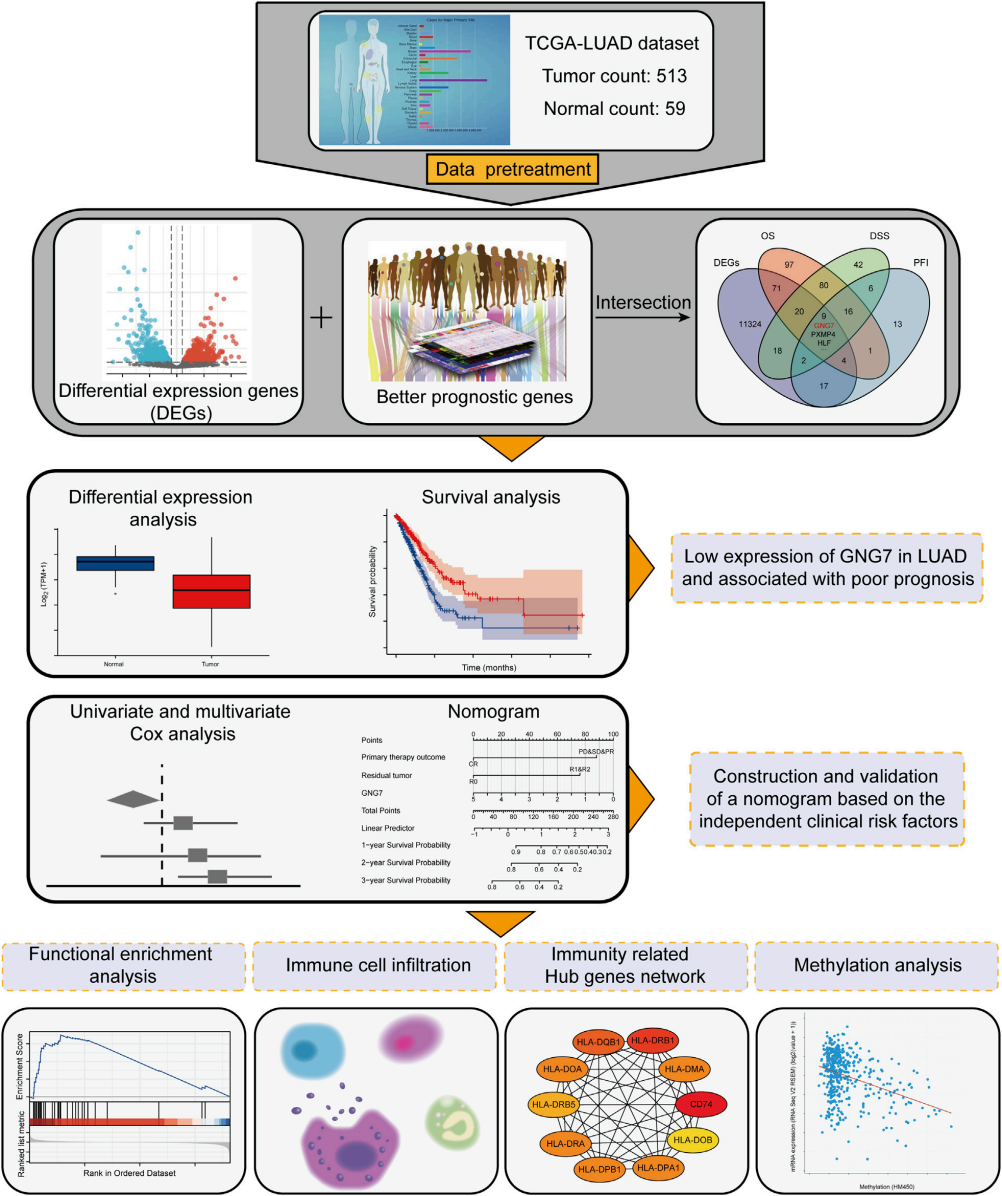
Workflow for screening of key genes and downstream analysis.

**FIGURE 2:**
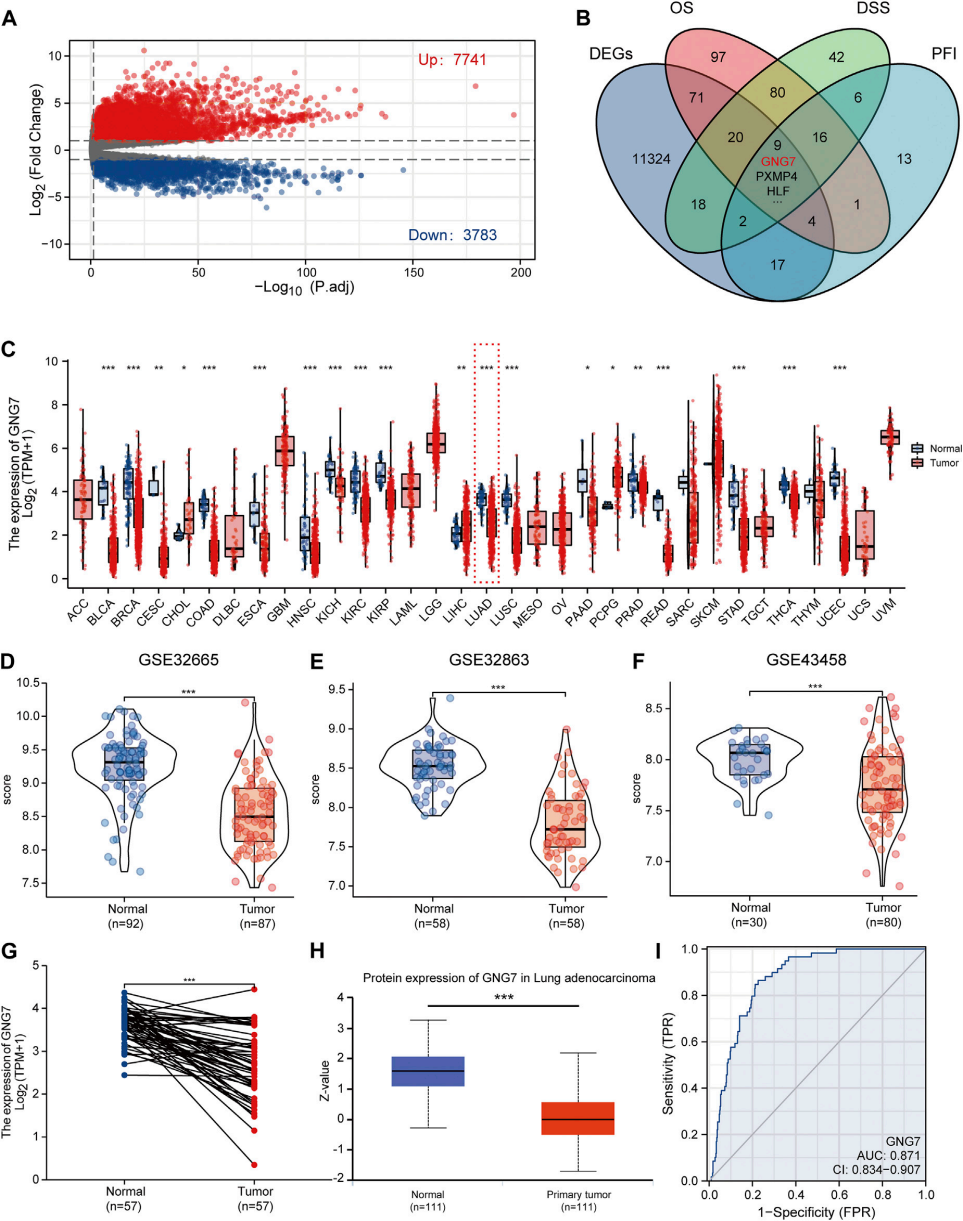
Screening of key regulators and identification of the differential expression of GNG7 in LUAD. **(A)** The volcanic map of the DEGs in LUAD. **(B)** A Venn diagram used to identify nine DEGs associated with LUAD prognostic indicators. **(C)** The GNG7 expression in different cancer from the TCGA database. **(D–F)** The GNG7 mRNA expression between LUAD and normal tissues based on data from GSE32665 **(D)**, GSE32863 **(E)** and GSE43458 **(F)** dataset. **(G)** The GNG7 mRNA expression between paired LUAD tumor tissues and adjacent normal tissues from the TCGA-LUAD dataset. **(H)** The GNG7 protein expression between LUAD and normal tissues from the UALCAN database. **(I)** A ROC curve to test the efficiency of GNG7 to identify LUAD from normal lung tissue. *p < 0.05, **p < 0.01, ***p < 0.001.

To find key regulators of LUAD, we conducted screening work based on differential expression analysis and prognostic analysis, and performed a series of analytical work based on the target gene. The workflow was shown in Figure 1 we applied the DESeq2 R package to screen for DEGs in LUAD based on TCGA-LUAD datasets. The results showed that there were 7741 up-regulated and 3783 down-regulated genes among the screened 11,524 DEGs (Figure 2A). Combined with further prognostic analysis, we found that among the DEGs, nine genes (HSD17B6, PXMP4, HLF, ADGRD1, CYP17A1, ESYT3, FCAMR, C11orf16, GNG7) were significantly and positively associated with LUAD prognostic indicators including OS, DSS and PFI (Figure 2B). Of note, although GNG7 has been reported to be differentially expressed in a variety of tumors, its roles in the initiation and progression of LUAD remain unclear. In the present study, we focused on GNG7 to explore the underlying mechanism and clinical significance in LUAD.

### G protein subunit gamma 7 expression is downregulated in lung adenocarcinoma

To elucidate the expression pattern of GNG7 in cancers, we first evaluated the expression of GNG7 in 33 types of cancers by a systematic analysis based on the TCGA databases. The results showed that GNG7 expression was significantly down-regulated in 17 different tumors, including Bladder Urothelial Carcinoma (BLCA), Breast invasive carcinoma (BRCA), Colon adenocarcinoma (COAD), Lung adenocarcinoma (LUAD) while it was significantly up-regulated in Cholangiocarcinoma (CHOL), Liver hepatocellular carcinoma (LIHC) and Pheochromocytoma and Paraganglioma (PCPG) (Figure 2C). Then, the low expression of GNG7 in LUAD was further validated by using three GEO datasets (GSE32665, GSE32863, GSE43458) (Figures 2D–F). The paired analysis got the similar results (Figure 2G). In addition, the decreased protein level of GNG7 was also observed in LUAD using the UALCAN database (Figure 2H). Moreover, ROC curve analysis was employed to analyze the distinguishing efficacy of GNG7 between LUAD tissue and normal tissue. The area under the curve (AUC) of GNG7 is 0.871, suggesting that GNG7 may be an ideal biomarker to distinguish LUAD from normal tissue (Figure 2I). Together, these results indicated that GNG7 is lowly expressed in LUAD which may be a potential diagnostic marker for LUAD.

### Association between clinicopathological characteristics and GNG7 expression in lung adenocarcinoma

**FIGURE 3:**
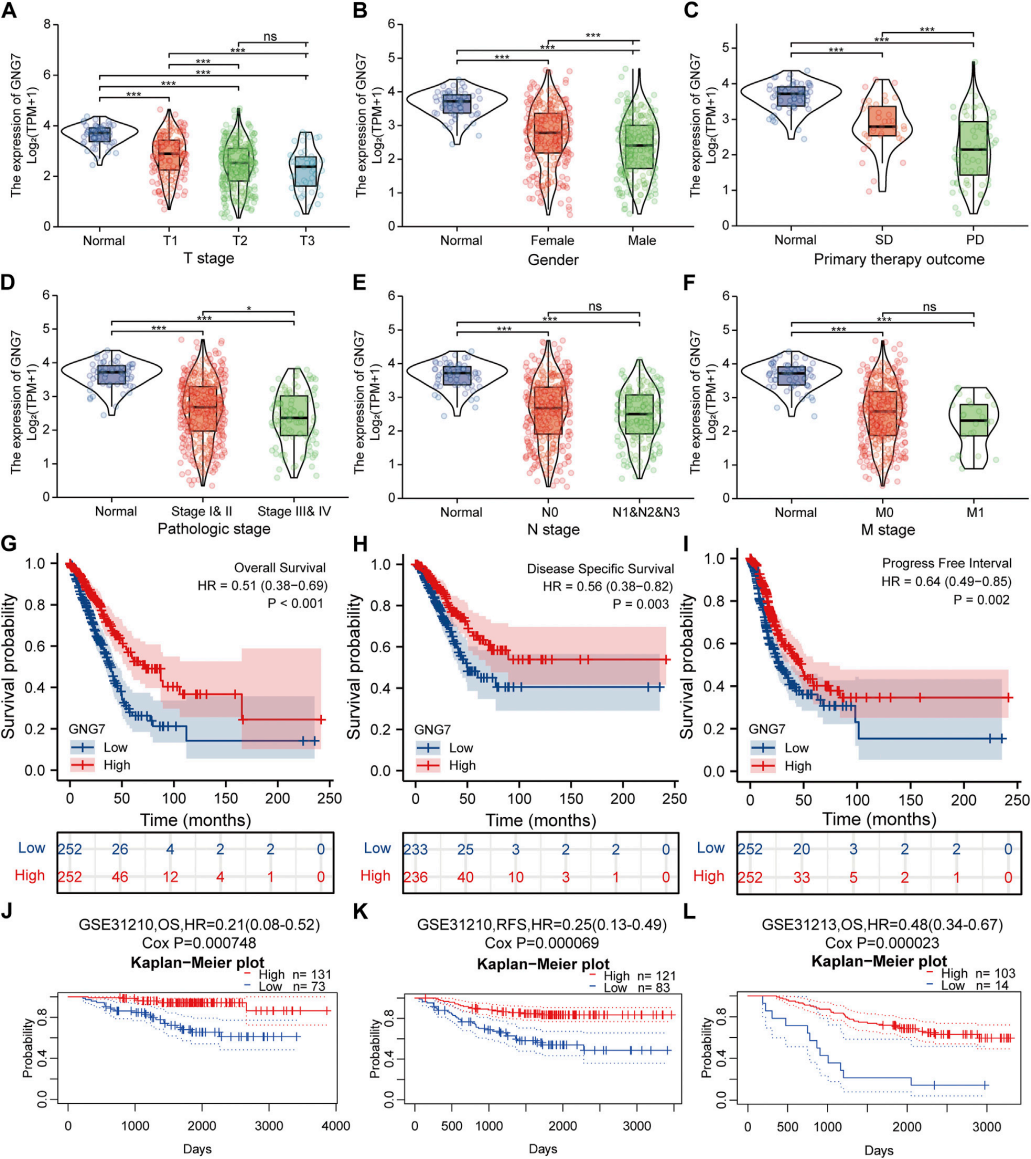
Correlation between GNG7 expression and clinicopathological features as well as the prognostic value of GNG7 in LUAD. **(A)** T stage. **(B)** Gender. **(C)** Primary therapy outcome. **(D)** Pathologic stage. **(E)** N stage. **(F)** M stage. ns, no significant difference, *p < 0.05, **p < 0.01, ***p < 0.001. **(G–I)** Survival curves of OS **(G)**, DSS **(H)**, and PFI **(I)** between GNG7-high and GNG7-low expression groups from the TCGA-LUAD dataset. **(J–L)** Kaplan-Meier survival curve analysis of OS **(J)** and RFS **(K)** in a LUAD cohort (GSE31210) as well as OS **(L)** in a LUAD cohort (GSE31213) from the PrognoScan database.

To clarify the correlation between the expression of GNG7 and clinicopathological variables, we collected data from the TCGA database on 535 patients with LUAD. After data preprocessing, the relationship between gene expression profiles and clinicopathological characteristics of 513 LUAD patients was shown in the baseline data table (Supplementary Table S1). The results showed that low expression of GNG7 was positively associated with high T stage, Gender (male sex), poor primary therapy outcome and high pathologic stage of LUAD, while there were no significant associations between GNG7 expression and the other clinical factors such as N stage and M stage (Figures 3A–F). In line with these findings, the logistics regression analysis also revealed that GNG7 expression was significantly associated with T stage (OR = 0.403, 95% CI: 0.274-0.589, *p* < 0.001), Pathologic stage (OR = 0.538, 95% CI: 0.377-0.766, *p* < 0.001), Primary therapy outcome (OR = 0.311, 95% CI: 0.133-0.707, *p* = 0.006) and Gender (OR = 0.502, 95% CI: 0.352-0.713, *p* < 0.001) (Table 1).

**TABLE 1:** GNG7 expression association with clinical pathological characteristics (logistic regression).

Characteristics	Total (N)	Odds Ratio (OR)	*p* value
T stage (T2&T3&T4 vs. T1)	510	0.403 (0.274−0.589)	***<0.001
N stage (N1&N2&N3 vs. N0)	501	0.700 (0.482−1.014)	0.060
M stage (M1 vs. M0)	369	0.493 (0.197−1.140)	0.110
Pathologic stage (Stage II&Stage III&Stage IV vs. Stage I)	505	0.538 (0.377−0.766)	***<0.001
Primary therapy outcome (PD vs. SD)	105	0.311 (0.133−0.707)	**0.006
Gender (Male vs. Female)	513	0.502 (0.352−0.713)	***<0.001
Race (Black or African American&White vs. Asian)	446	2.701 (0.576−19.003)	0.238
Age (>65 vs. <=65)	494	1.050 (0.738−1.496)	0.785
Residual tumor (R1&R2 vs. R0)	361	0.565 (0.191−1.519)	0.271
Anatomic neoplasm subdivision (Right vs. Left)	498	0.957 (0.669−1.371)	0.812
Anatomic neoplasm subdivision2 (Peripheral Lung vs. Central Lung)	189	1.022 (0.555−1.894)	0.943
number_pack_years_smoked (>=40 vs. <40)	351	0.863 (0.567−1.312)	0.491
Smoker (Yes vs. No)	499	0.629 (0.378−1.035)	0.070

*p < 0.05; **p < 0.01; ***p < 0.001.

### Significance of G protein subunit gamma 7 in clinical prognosis of lung adenocarcinoma and clinical subgroup analysis

We utilized data from the TCGA database to investigate the prognostic significance of GNG7 in LUAD. Kaplan-Meier survival analysis based on the TCGA-LUAD dataset revealed that low expression of GNG7 was associated with poor OS (HR = 0.51, 95% CI: 0.38-0.69, *p* < 0.001), DSS (HR = 0.56, 95% CI: 0.38-0.82, *p* = 0.003) and PFI (HR = 0.64, 95% CI: 0.49-0.85, *p* = 0.002) (Figures 3G–I). To further validate the prognostic value of GNG7 in LUAD, we utilized the PrognoScan database for further study. We included two of the GSE datasets (GSE31210 and GSE13213) in our analysis, where low GNG7 expression was significantly associated with the poorer prognosis (OS, HR = 0.21, 95% CI: 0,08-0.52, Cox *p* = 0.000748; RFS, HR = 0.25, 95% CI: 0,13-0.49, Cox *p* = 0.000069 in the GSE31210 dataset; OS, HR = 0.48, 95% CI: 0,34-0.67, Cox *p* = 0.000023 in the GSE13213 dataset) (Figures 3J–L).

Moreover, the univariate Cox regression analysis model showed that GNG7 expression level was significantly associated with OS (HR: 0.702; 95% CI: 0.599-0.822; *p* < 0.001) similar to T stage, N stage, M stage and Pathologic stage as well Primary therapy outcome and Residual tumor. Meanwhile, the multivariate Cox regression analysis also revealed that low expression of GNG7, similar to Primary therapy outcome and Residual tumor, was an independent risk factor for the prognosis of LUAD patients (Table 2). Collectively, these results suggest that low expression of GNG7 independently predicts poor prognosis for patients with LUAD.

**TABLE 2:** Univariate analysis and multivariate analysis of the correlation between clinicopathological characteristics and OS in LUAD.

Characteristics	Total (N)	Univariate analysis		Multivariate analysis	
		Hazard ratio (95% CI)	*p* value	Hazard ratio (95% CI)	*p* value
T stage (T2&T3&T4 vs. T1)	501	1.668 (1.184−2.349)	**0.003	1.116 (0.629−1.978)	0.708
N stage (N1&N2&N3 vs. N0)	492	2.606 (1.939−3.503)	***<0.001	1.689 (0.758−3.764)	0.200
M stage (M1 vs. M0)	360	2.111 (1.232−3.616)	**0.007	1.698 (0.674−4.280)	0.262
Pathologic stage (Stage II&Stage III&Stage IV vs. Stage I)	496	2.975 (2.188−4.045)	***<0.001	1.109 (0.471−2.610)	0.812
Primary therapy outcome (PD&SD&PR vs. CR)	419	2.818 (2.004−3.963)	***<0.001	3.662 (2.217−6.049)	***<0.001
Gender (Male vs. Female)	504	1.060 (0.792−1.418)	0.694		
Race (White vs. Asian&Black or African American)	446	1.422 (0.869−2.327)	0.162		
Age (>65 vs. <=65)	494	1.228 (0.915−1.649)	0.171		
Residual tumor (R1&R2 vs. R0)	352	3.973 (2.217−7.120)	***<0.001	3.670 (1.503−8.964)	**0.004
Anatomic neoplasm subdivision (Right vs. Left)	490	1.024 (0.758−1.383)	0.878		
Anatomic neoplasm subdivision2 (Peripheral Lung vs. Central Lung)	182	0.913 (0.570−1.463)	0.706		
Number pack years smoked (>=40 vs. <40)	345	1.038 (0.723−1.490)	0.840		
Smoker (Yes vs. No)	490	0.887 (0.587−1.339)	0.568		
GNG7	504	0.702 (0.599−0.822)	***<0.001	0.727 (0.561-0.943)	*0.016

*p < 0.05; **p < 0.01; ***p < 0.001.

**FIGURE 4:**
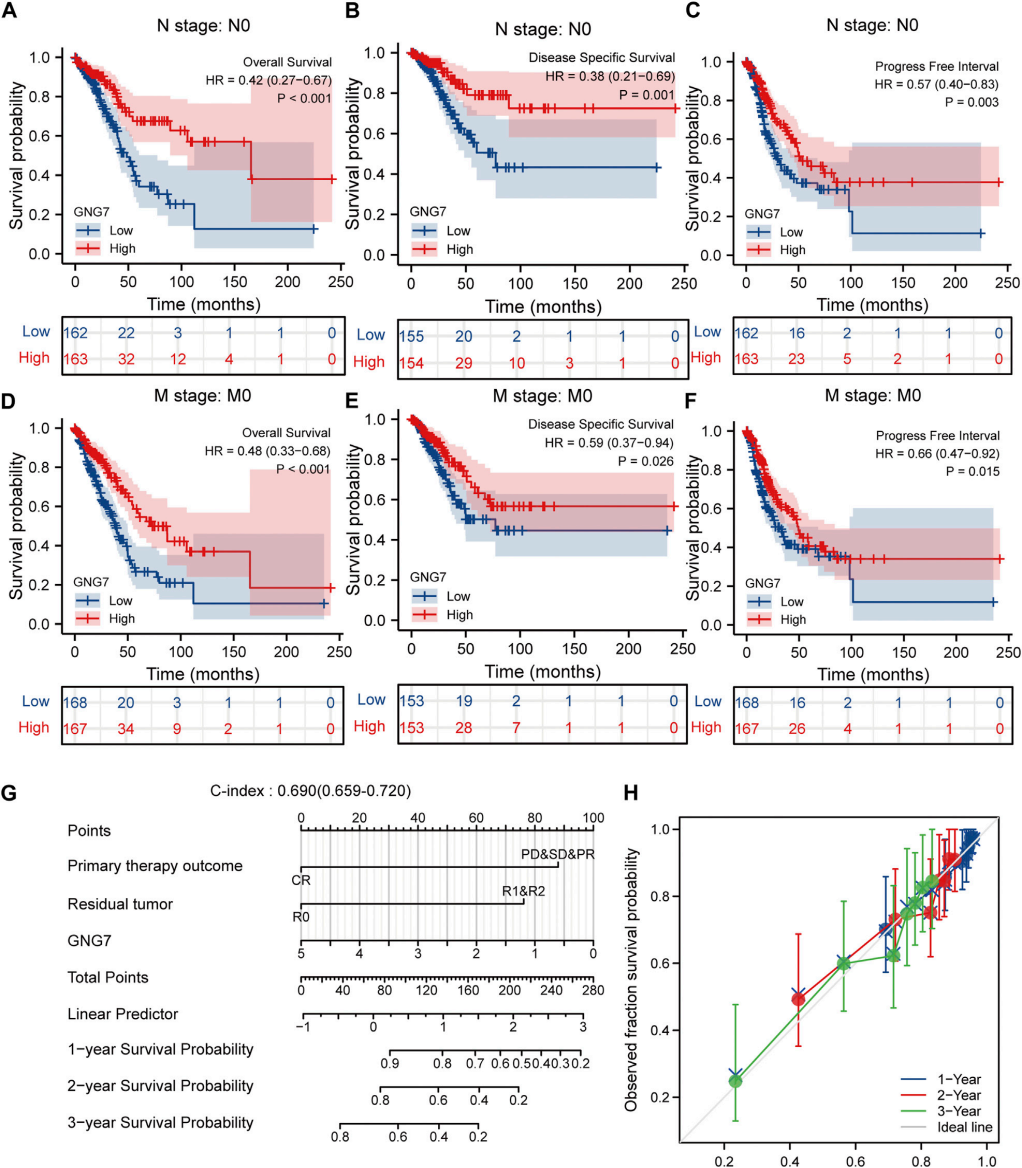
Subgroup analysis and the construction of a nomogram based on GNG7 expression. **(A–C)** The Kaplan-Meier curves of OS **(A)**, DSS **(B)**, and PFI **(C)** between GNG7-high and -low expression patients with LUAD in N0 stage. **(D–F)** The Kaplan-Meier curves of OS **(D)**, DSS **(E)**, and PFI **(F)** between GNG7-high and -low expression patients with LUAD in M0 stage. **(G)** A nomogram that integrates GNG7 and other independent prognostic factors in LUAD from TCGA data. **(H)** The calibration curve of the nomogram. OS, overall survival; DSS, disease specific survival; PFI, progress free interval; LUAD, lung adenocarcinoma.

Given that multivariate Cox regression analysis identified low expression of GNG7 as an independent risk factor, we investigated the potential prognostic value of GNG7 in LUAD patients with different clinical subgroups. As shown in Figures 4A–C, low expression of GNG7 was associated with poor prognosis in stage N0, including OS (HR = 0.42, 95% CI: 0.27-0.67, *p* < 0.001), DSS (HR = 0.38, 95% CI: 0.21-0.69, *p* = 0.001) and PFI (HR = 0.57, 95% CI: 0.40-0.83, *p* = 0.003). However, there was no statistically significant correlation between GNG7 expression and prognosis in the N1&N2&N3 stage (*p* > 0.05) (Supplementary Figures S1A–C). In addition, low GNG7 expression was significantly associated with poor prognosis in LUAD patients in M0 stage, including OS (HR = 0.48, 95% CI: 0.33-0.68, *p* < 0.001), DSS (HR = 0.59, 95% CI: 0.37-0.94, *p* = 0.026), PFI (HR = 0.66, 95% CI: 0.47-0.92, *p* = 0.015) (Figures 4D–F). Nevertheless, no significant association was shown between GNG7 expression and prognosis in LUAD patients in the M1 stage (Supplementary Figures S1D–F). These results suggest that low expression of GNG7 is positively associated with the poor prognosis of LUAD patients without lymph node invasion and distal metastasis.

### Construction and validation of a nomogram based on the independent clinical risk factors

To provide a quantitative approach to predicting the prognosis of LUAD patients, we constructed a prognostic nomogram to predict individual survival probability based on the expression levels of GNG7 and other independent clinical risk factors (Figure 4G). The calibration curve of the nomogram showed that the established lines of 1-, 2-, and 3-y survival highly matched the ideal line (the 45-degree line) (Figure 4H). In addition, the C-index of the prediction model reached 0.690 (0.659–0.720), indicating that the model had a reliable potential to predict the OS of LUAD patients. In addition, ROC curve analysis based on the three time points of 1-, 2-, and 3-Year showed that the Area under the curve (AUC) of this prediction model was higher than the AUC of the two-by-two model consisting of independent prognostic factors screened from multivariate Cox regression, indicating the superiority of the model (Supplementary Figures S2A–D). On the basis of the median risk score, patients were divided into a high-risk score group and a low-risk score group. Survival curve analysis revealed that the high-risk group had a significantly poorer prognosis compared to the low-risk group (HR = 2.59, 95% CI: 2.59 (1.72–3.89), *p* < 0.001) (Supplementary Figure S2E). Additionally, the risk curve indicated that the high-risk score group had higher mortality and worse prognosis than the low-risk score group (Supplementary Figure S2F).

### Functional enrichment and pathway analysis of G protein subunit gamma 7-associated differentially expressed genes in lung adenocarcinoma

**FIGURE 5:**
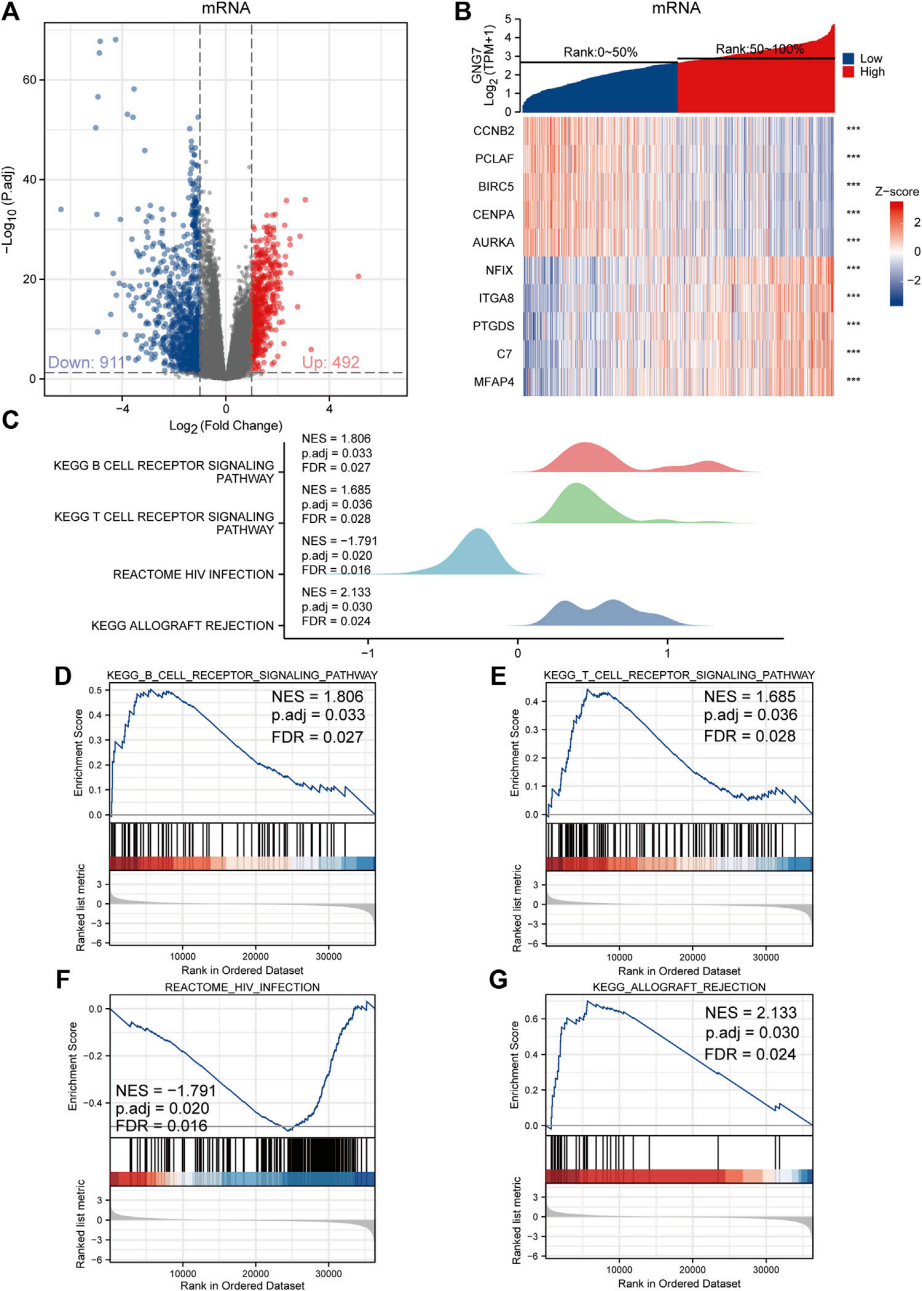
Functional annotation of differentially expressed genes (DEGs) regulated by GNG7 in LUAD. **(A,B)** Based on the median GNG7 expression level, LUAD patients from the TCGA-LUAD dataset were stratified into GNG7-high and GNG7-low groups. Expression profiles of mRNAs in two groups are presented by volcano plots **(A)** and heatmaps **(B)**. **(C)** Pathway enrichment plots from GSEA. **(D–G)** The B cell receptor signaling pathway **(D)**, T cell receptor signaling pathway **(E)**, HIV infection **(F)**, and allograft rejection **(G)** were positively correlated to GNG7 expression. TCGA, the cancer genome atlas; LUAD, lung adenocarcinoma; NES, normalized enrichment score; FDR, false discovery rate.

To investigate the biological functions and signaling pathways associated with GNG7, we examined the DEGs between GNG7-high and GNG7-low patients which were stratified based on the median GNG7 expression. Resultantly, 1403 mRNAs (492 upregulated and 911 downregulated), 962 lncRNAs (256 upregulated and 706 downregulated), and 21miRNAs (18 upregulated and 3 downregulated) were differently expressed in GNG7-high patients compared to GNG7-low ones (Figure 5A, Supplementary Figures S3A,C). Relative expression values of some representative DEGs between the two cohorts were shown in the form of heatmaps (Figure 5B, Supplementary Figures S3B,D). Strikingly, pathway enrichment analysis showed that the DEGs were most strongly enriched in the B cell receptor signaling pathway, T cell receptor signaling pathway and HIV infection allograft rejection which are highly related to the cellular immune response (Figures 5C–G). These data suggested that GNG7 may play an important role in regulating the tumor immune microenvironment of LUAD.

### Correlation analysis between the expression of G protein subunit gamma 7 and immune cell infiltration in lung adenocarcinoma

**FIGURE 6:**
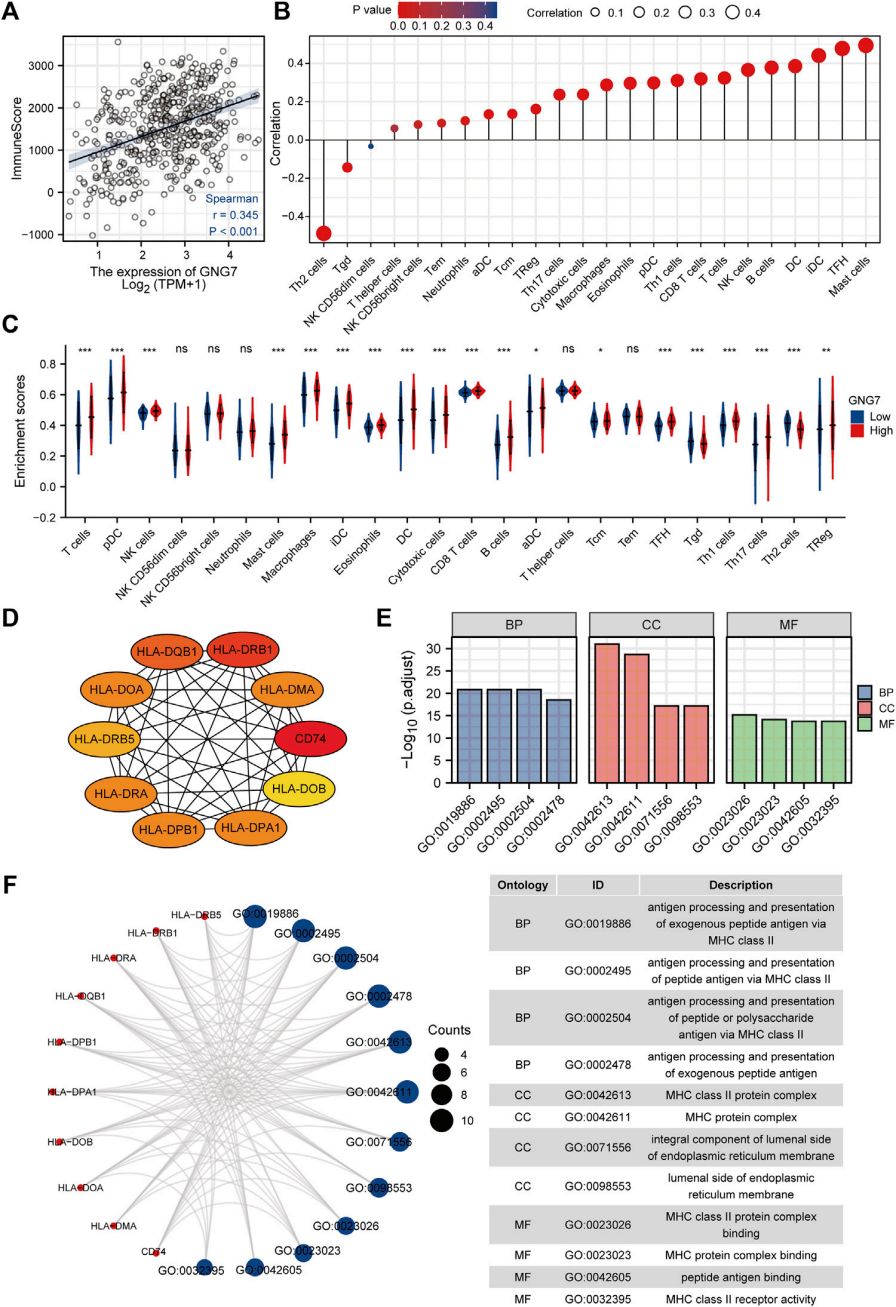
Correlation of immune cell infiltration and GNG7 expression in LUAD patients. **(A)** Relationship between immune scores and GNG7 expression levels in LUAD. **(B)** Relationships between infiltration levels of 24 immune cell types and GNG7 expression profiles by Spearman’s analysis. **(C)** Comparison of the immune infiltration level of 24 immune cell types between GNG7-high and GNG7-low groups. LUAD, lung adenocarcinoma; DCs, dendritic cells; aDCs, activated DCs; iDCs, immature DCs; pDCs, plasmacytoid DCs; Th, T helper cells; Th1, type 1 Th cells; Th2, type 2 Th cells; Th17, type 17 Th cells; Treg, regulatory T cells; Tgd, T gamma delta; Tcm, T central memory; Tem, T effector memory; Tfh, T follicular helper; NK, natural killer. **(D)** The top10 hub genes calculated with the MCC algorithm by cytoHubb. **(E)** The top 4 enriched GO terms of BP, CC and MF categories of the GO enrichment analysis. **(F)** The network including the hub genes and the enriched GO terms. BP, biological process; CC, cellular component; MF, molecular function.

As reported, tumor-associated immune cell infiltration has a close relationship with tumor development and the prognosis of patients. Then, we utilized the ESTIMATE algorithm to assess the correlation between GNG7 and the abundance of immune cell infiltration in LUAD. The results revealed that GNG7 expression was positively correlated with the abundance of immune cell infiltration in LUAD (Figure 6A). Specifically, further Spearman correlation analysis showed that among 24 immune cell subpopulations, GNG7 expression was positively correlated with most immune cell subsets, including Mast cell, DC, B cells, and CD8^+^ T cells, but negatively correlated with Th2 and Tgd cells (Figure 6B). Consistently, the ssGSEA analysis demonstrated that the infiltration levels of most of the immune cell subsets such as Mast cells, pDCs, B cells, NK cells and CD8^+^ T cells were remarkably increased in LUAD patients with GNG7 high expression compared to those with GNG7 low expression (Figure 6C). In keeping with this finding, GNG7 was significantly correlated with most immune markers of different immune cells, including CD8^+^T cell, B cell, Neutrophils, and Dendritic cells (Supplementary Table S2). Moreover, by correlation analysis, we identified immune-related genes (IRG) co-expressed with GNG7 and constructed a PPI network (Supplementary Figure S4A). We screened the top10 of hub genes and showed the correlation between these 10 genes and GNG7 in the form of scatter plots (Figure 6D, Supplementary Figures S4B–K). Additionally, we performed GO enrichment analysis to investigate the possible involvement of GNG7 in the immune response. The terms identified in the BP category showed that aberrantly expressed GNG7 was associated with antigen processing and presentation *via* MHC class II(MHCII), while in the CC category, the hub genes were significantly enriched in the MHCII protein complex and endoplasmic reticulum-related terms. Furthermore, the MF category revealed significant enrichment in GO terms related to the MHCII protein complex binding, MHCII receptor activity and peptide antigen binding, etc (Figures 6E,F). Together, these results suggest that GNG7 may contribute to the remodeling of the immune microenvironment in LUAD through promoting the infiltration of a variety of tumor-associated immune cells and influencing antigen presentation.

### G protein subunit gamma 7 high expression with high B cell infiltration predicts a better prognosis of lung adenocarcinoma patients

**FIGURE 7:**
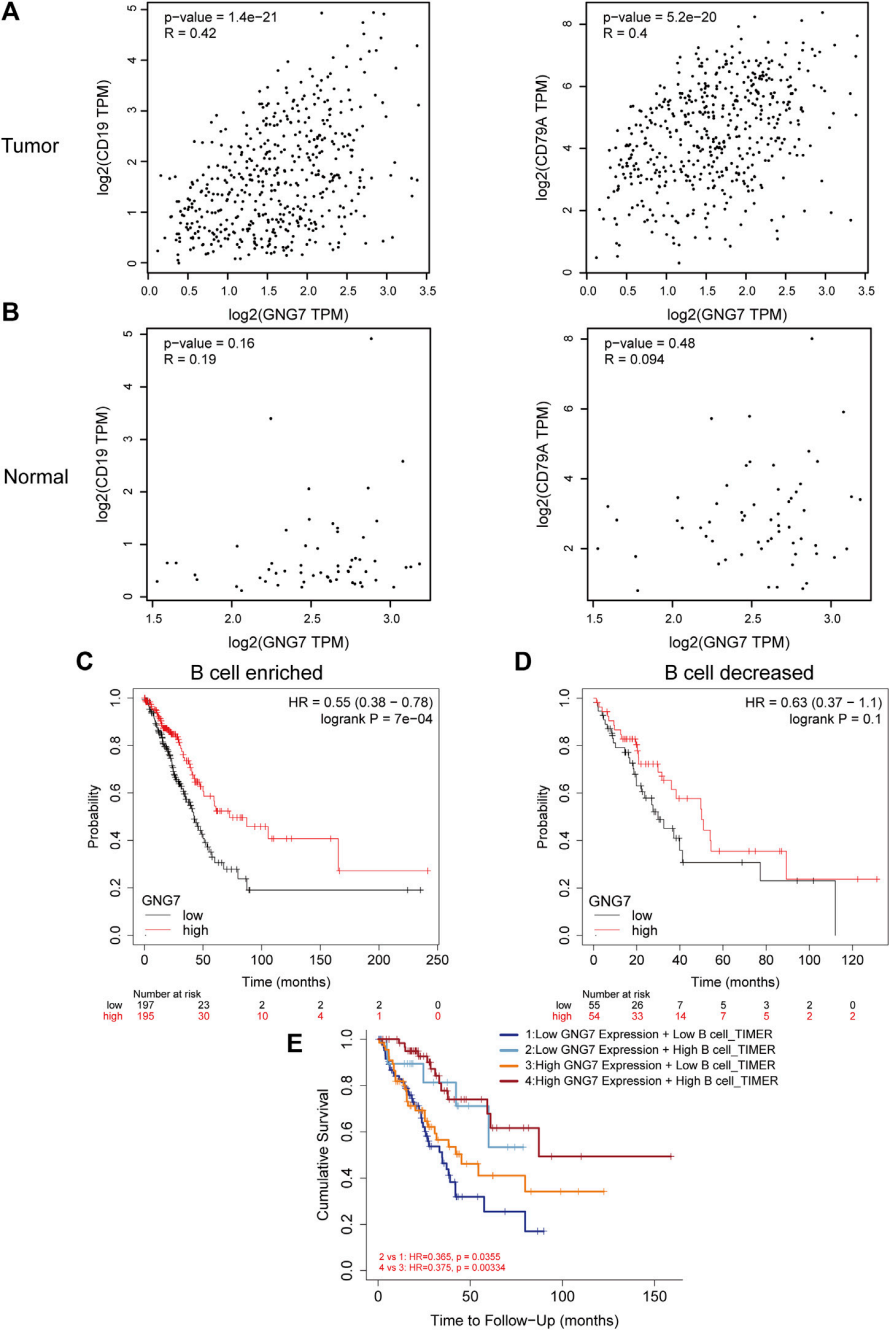
Correlation between GNG7 and B cell immune infiltration and prognostic analysis in LUAD. **(A)** The correlation between the expression of GNG7 and B cell markers CD19 (left) as well CD79A (right) in LUAD tissues. **(B)** The correlation between the expression of GNG7 and B cell markers CD19 (left) as well CD79A (right) in normal lung tissues. **(C)** Kaplan-Meier survival curves of OS in LUAD based on GNG7 expression in the enriched B cells groups. **(D)** Kaplan-Meier survival curves of OS based on GNG7 expression in the decreased B cells groups. **(E)** Kaplan-Meier survival curves of OS in LUAD based on B cell infiltration level in GNG7-high and -low expression patients. LUAD, lung adenocarcinoma; OS, overall survival.

Of the infiltrated immune cells increased in LUAD with GNG7 high expression, B cell infiltration attracts our attention as the relatively less knowledge of this cell type in tumor immunotherapy currently. Our results showed that GNG7 was closely associated with the level of B cell infiltration in LUAD (Supplementary Figure S5A). Specifically, the level of B cell infiltration was significantly elevated in the GNG7 high expression group compared with that in the GNG7 low expression group (Supplementary Figure S5B). In addition, we respectively investigated the correlation of GNG7 with B cells in the tumor and normal tissues. Strikingly, GNG7 showed a strong positive correlation with the B cell marker genes CD19 and CD79A in LUAD tissues (Figure 7A). In contrast, the correlation of GNG7 with the B cell markers CD19 and CD79A did not reach statistical significance in normal tissues (Figure 7B). These results suggest that GNG7 expression may promote B cell infiltration in the context of LUAD. Meanwhile, through KM plot database analysis, we found that patients with high GNG7 expression tended to predict a better prognosis in the B cell enriched group but not in the B cell decreased group (Figures 7C,D). Such finding was further corroborated by the analysis using the TIMER2.0 database, implying that high GNG7 expression corresponded to a better prognosis for LUAD patients in the context of enriched B cell infiltration. Furthermore, we found that high infiltration levels of B cells in the presence of consistent levels of GNG7 expression corresponded to a good prognosis in patients with LUAD (Figure 7E). Taken together, it is reasonable to suggest that GNG7 may have improved patient prognosis by promoting B cell infiltration.

### G protein subunit gamma 7 dysregulation is associated with aberrant DNA methylation

**FIGURE 8:**
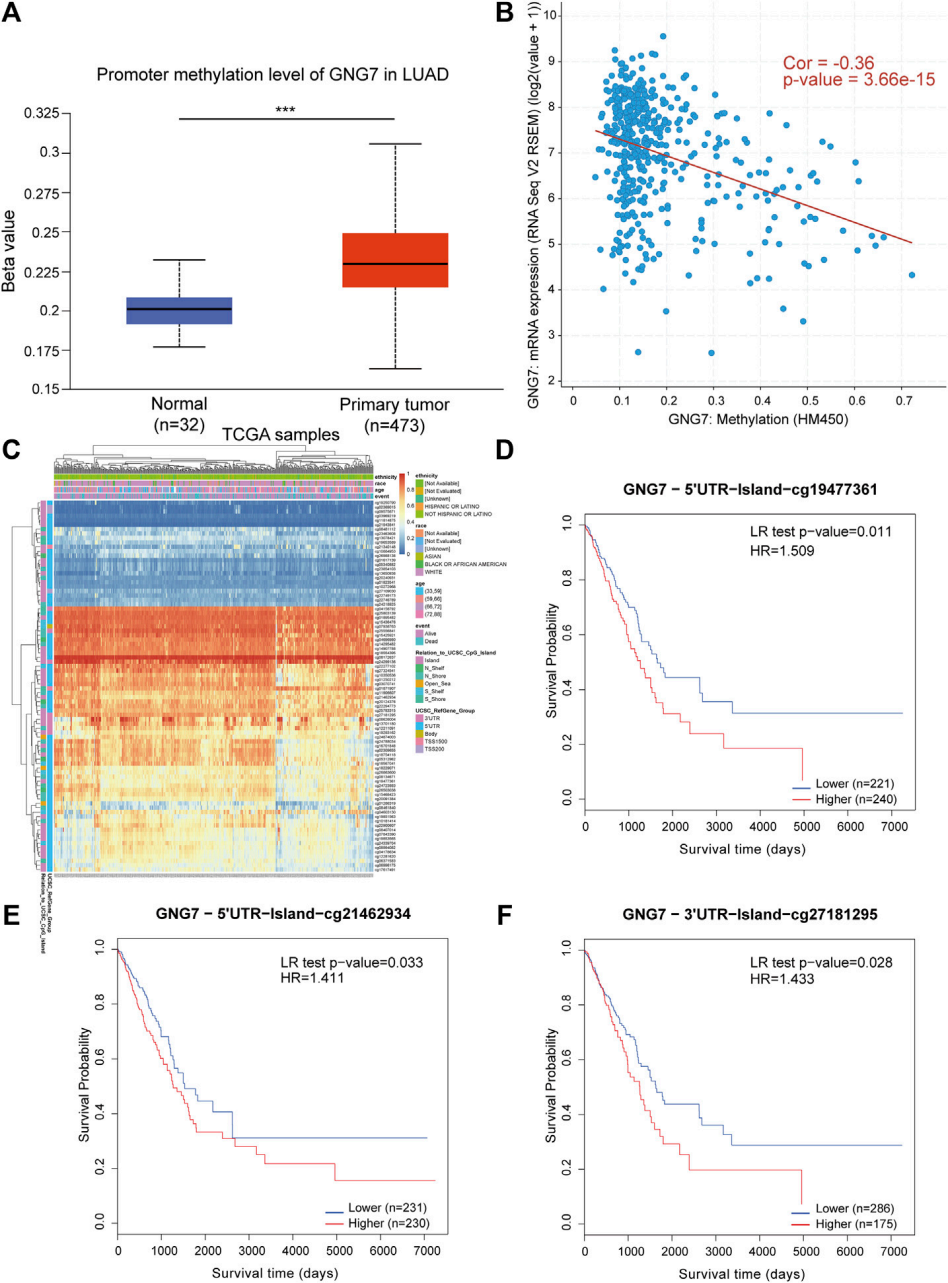
DNA methylation levels of GNG7 and its prognostic value in LUAD. **(A)** The promoter methylation level of GNG7 in normal tissues and primary LUAD tissues by the UALCAN database. **(B)** The correlation between GNG7 methylation and its expression level. **(C)** The heatmap of DNA methylation at CpG sites in the GNG7 gene by the MethSurv database. **(D–F)** Kaplan-Meier survival curves of OS based on methylation at GpG sites of cg19477361 **(D)**, cg21462934 **(E)** and cg27181295 **(F)** in LUAD.

Considering the importance of DNA methylation in regulating gene expression, we tested whether aberrant DNA methylation occurs in *GNG7* gene in LUAD. By analyzing the data from the UALCAN database, we found that the methylation level of GNG7 was significantly higher in the tumor group compared to that in the normal group (Figure 8A). Next, the correlation analysis based on the cBioPortal database showed that GNG7 expression was significantly negatively correlated with methylation (Figure 8B). To further investigate the methylation of GNG7 in LUAD, we analyzed the methylation levels of different CpG sites of GNG7 in LUAD patients using the MethSurv database and presented them in the form of heat maps (Figure 8C). The results revealed that several CpG sites of GNG7 exhibited high methylation in LUAD patient samples, including cg19477361, cg21462934, and cg27181295. Prognostic analysis showed that the above CpG sites with highly methylated levels were associated with poor prognosis in LUAD (Figures 8D–F). These results suggest that the low expression of GNG7 in LUAD may be partly due to the methylation modification of the abovementioned CpG sites and plays a key role in tumor progression.

## Discussion

As one of the most common malignancies worldwide, the prognosis of LUAD patients remains very gloomy due to the lack of effective biomarkers for early diagnosis and effective treatment for advanced patients. Thus, intense research has been focused on deciphering the pathogenesis and searching for effective diagnostic and therapeutic approaches as well as prognostic markers to improve the prognosis of patients with LUAD in the last several years. Indeed, a growing number of potential biomarkers for LUAD have been identified, such as PPP1R14D, lncRNA-Ac068228, IFITM1, and so on (Koh et al., 2019; Jiang et al., 2022; Tian Y. et al., 2022). However, most of these biomarkers are associated with the increase in cell numbers resulting from cell division (cell proliferation), programmed cell death (apoptosis), and tumor angiogenesis, while few with tumor immune microenvironment. Accumulating evidence has shown that not only the characteristics of tumor cells but also the tumor microenvironment, especially Tumor infiltrating immune cells (TIICs), plays critical roles in the tumorigenesis and progression of LUAD (Hinshaw and Shevde, 2019). In recent years, with the further understanding of the mechanism of tumor immune infiltration, tumor immunotherapies, such as the immune checkpoint inhibitors (ICIs), have had a revolutionary impact on the treatment of LUAD (Bagchi et al., 2021). However, only a small percentage of patients achieved a durable immune response after treatment. The mechanisms of LUAD development are far from being elucidated. It is of great necessity to further clarify the molecular basis of LUAD and explore contributing factors as well as sensitive diagnosis and prognosis biomarkers of immunotherapy response to improve patient outcomes (Wu and Shih, 2018; Singh et al., 2020).

In the current study, we performed comprehensive bioinformatics analyses to explore the potential key molecules involved in the development of LUAD. Through screening and identification, GNG7 was demonstrated to be lowly expressed in LUAD and had a good diagnostic performance. In addition, low expression of GNG7 was positively associated with the poor clinicopathological characteristics such as poor primary therapy outcome and high pathologic stage of LUAD, implying the tumor suppressive roles of GNG7 in LUAD.

As a subunit of heterotrimeric G protein, GNG7 has been reported to be tightly related to carcinogenesis. GNG7 is frequently downregulated in various cancers including pancreatic cancer, esophageal cancer and clear cell renal cell carcinoma (Shibata et al., 1998; Ohta et al., 2008; Xu et al., 2019). It is worth noting that GNG7 has been identified as one of the hub genes in an eight-gene prognostic signature model and a four-gene panel predicting overall survival for LUAD (Li C. et al., 2020; Ma et al., 2021). However, the role of GNG7 as an independent prognostic factor in LUAD has not been fully elucidated. Here, our study based on GNG7 is similar to, but more distinctive from, the newly identified biomarkers for LUAD in the latest literatures. In our study, we demonstrated GNG7 as an independent prognostic risk factor in LUAD (Supplementary Table S4) (Wan et al., 2021; Tian W. et al., 2022; Zhang et al., 2022; Zhou et al., 2022). Moreover, we constructed a nomogram combined with other clinical independent prognostic risk factors to predict the prognosis of LUAD patients reliably.

Recent studies have reported that GNG7 inhibited the progression of LUAD by inhibiting E2F1 and Hedgehog signaling, but the exact mechanism by which it regulates the development of LUAD is largely unknown (Zhao et al., 2021; Zheng et al., 2021). Our GSEA and GO enrichment analysis found that GNG7 may be involved in regulating the TME of LUAD and antigen processing and presentation *via* MHCII. Especially, high expression of GNG7 corresponded to increased infiltration level of several immune cells including B cells. Over the past decades, intense research has focused on the roles of T cells in immune regulation in the TME (Joyce and Fearon, 2015). More recently, there is increasing evidence supporting a critical role for B cells in tumor immunology (Bruno, 2020; Cabrita et al., 2020; Helmink et al., 2020). However, there is limited understanding of the biological contributors to the B cell infiltration in the TME. As the key immune cell in humoral immunity, B cells express a large number of MHCII molecules and are important antigen-presenting cells. In the present study, we found that GNG7 may be involved in regulating MHCII-mediated antigen processing and presentation. Furthermore, we found that GNG7 expression may promote B cell infiltration as evidenced by that low GNG7 expression was negatively correlated with B cell infiltration. Strikingly, the results of the prognostic analysis indicated patients with high GNG7 expression tended to predict a better prognosis in the context of enriched B cell infiltration and high infiltration levels of B cells regardless of GNG7-low or GNG7-high expression corresponded to a good prognosis in patients with LUAD. Our results indicated that GNG7 may exert its tumor suppressive roles in LUAD by promoting B cell infiltration and GNG7 expression together with B cell infiltration may be a powerful predictive signature for prognosis and immunotherapy response in LUAD, although the detailed function and mechanism need further in-depth investigation both *in vitro* and *in vivo*.

Finally, in this study, we further explored the mechanism of GNG7 low expression in LUAD. To our knowledge, other than miR-19b-3p which was reported to target GNG7 directly and significantly decrease the mRNA level of GNG7, little is known about the mechanism of GNG7 dysregulation in LUAD (Zhao et al., 2021). Given that DNA methylation of CpG islands is known to be a repressive mark of gene expression, we assessed the methylation level of GNG7 in LUAD (Kulis and Esteller, 2010). As expected, elevated methylation level of GNG7 was observed in tumor tissues which may be responsible for the low expression of GNG7 in LUAD. Interestingly, hypermethylation of GNG7 was associated with poor prognosis in patients with LUAD, which is consistent with the prognostic value of GNG7 mRNA expression. These results suggest the importance of DNA methylation in regulating GNG7 expression in LUAD.

In summary, this study revealed for the first time that GNG7 may be involved in regulating the immune microenvironment in LUAD and influence tumor development and patient prognosis at least partly by regulating the B cell infiltration. GNG7 may be not only a potential diagnostic biomarker for LUAD but also a promising predictive signature for prognosis and immunotherapy response for patients with LUAD. Nevertheless, as data from this study were mainly obtained from open databases, more LUAD patient samples are needed to confirm the clinical prognostic value of GNG7. Moreover, the effects of GNG7 on immune cell recruitment and infiltration as well as immunotherapy response are needed to be investigated deeply at the cellular and molecular levels and in future clinical trials.

## Data Availability

Publicly available datasets were analyzed in this study. This data can be found here: TCGA database: https://portal.gdc.cancer.gov/ and GEO database: https://www.ncbi.nlm.nih.gov/geo/.
