# Supplementary material for: Comprehensive analysis to identify GNG7 as a prognostic biomarker in lung adenocarcinoma correlating with immune infiltrates
Source: Front Genet. 2022 Sep 9;13:984575. doi: 10.3389/fgene.2022.984575 (PMC9500342; doi:10.3389/fgene.2022.984575)
Supplement: Supplementary file 1 [file DataSheet4.docx]

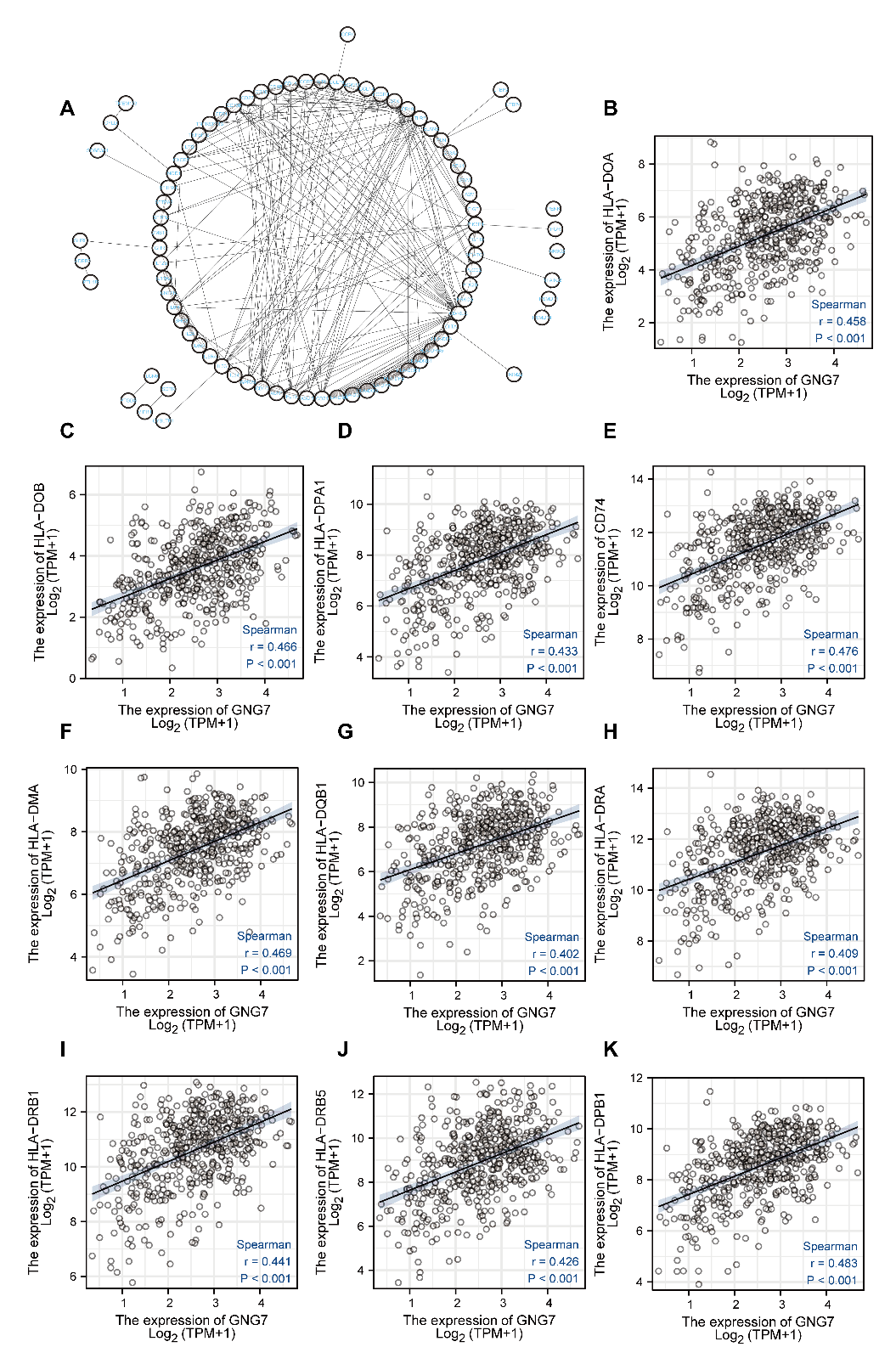


**Supplementary Figure 4. PPI (protein-protein interaction) network and the correlation between hub genes and GNG7 (A)** PPI network of immune-related genes (IRG) associated with GNG7. **(B-K)** Scatter plot of the correlation between hub gene and GNG7. (B) HLA-DOA. (C) HLA-DOB. (D) HLA-DPA1. (E) CD74. (F) HLA-DMA. (G) HLA-DQB1. (H) HLA-DRA. (I) HLA-DRB1. (J) HLA-DRB5. (K) HLA-DPB1.
