# Supplementary material for: Comprehensive analysis to identify GNG7 as a prognostic biomarker in lung adenocarcinoma correlating with immune infiltrates
Source: Front Genet. 2022 Sep 9;13:984575. doi: 10.3389/fgene.2022.984575 (PMC9500342; doi:10.3389/fgene.2022.984575)
Supplement: Supplementary file 2 [file DataSheet3.docx]

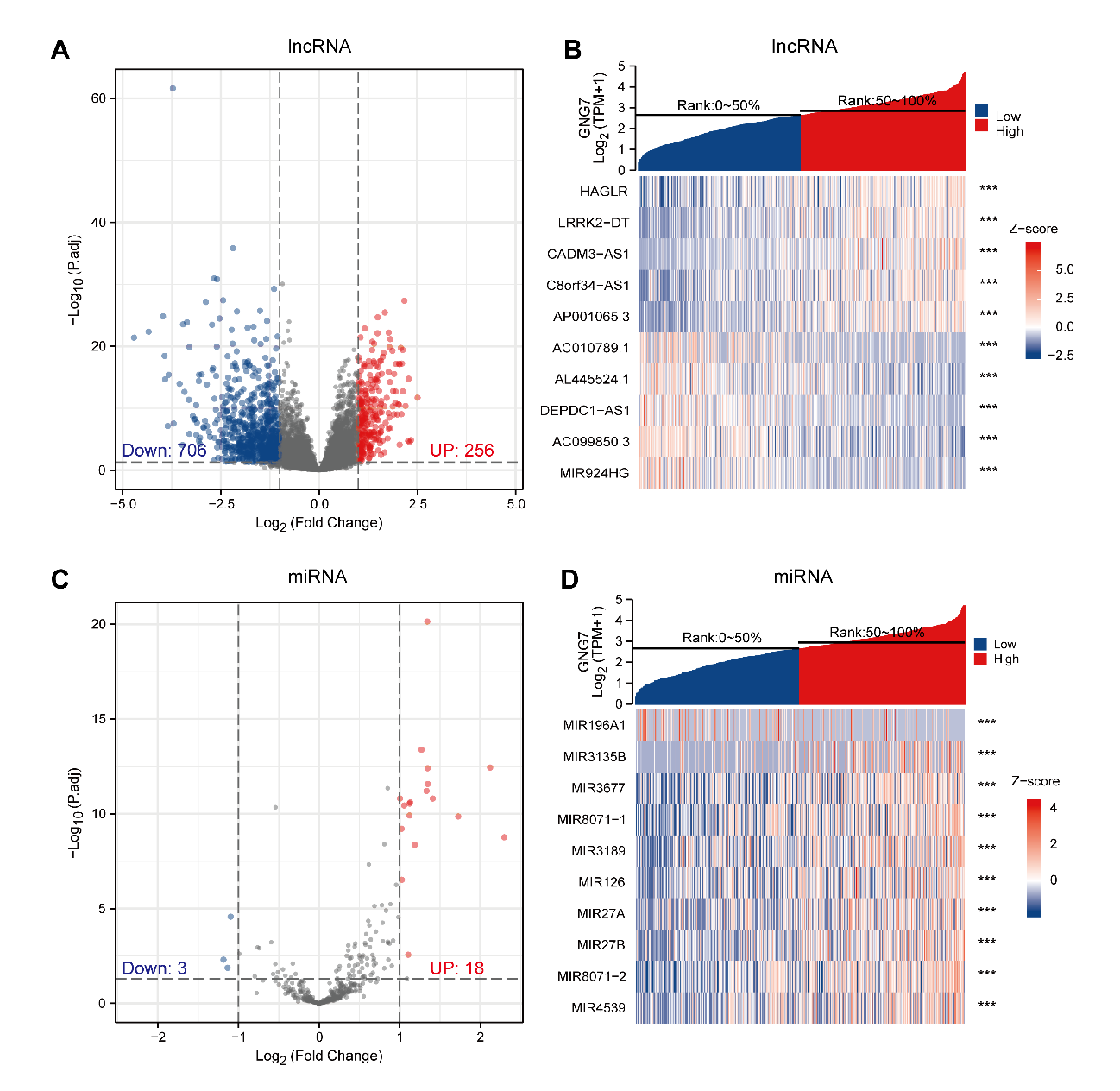


**Supplementary Figure 3. Differential non-coding RNA expression profiles in LUAD patients stratified by GNG7 levels.** **(A-B)** Expression profiles of lncRNAs in two groups are presented by volcano plots (A) and heatmaps (B). **(C-D)** Expression profiles of miRNAs in two groups are presented by volcano plots (C) and heatmaps (D).
