# Supplementary material for: Comprehensive analysis to identify GNG7 as a prognostic biomarker in lung adenocarcinoma correlating with immune infiltrates
Source: Front Genet. 2022 Sep 9;13:984575. doi: 10.3389/fgene.2022.984575 (PMC9500342; doi:10.3389/fgene.2022.984575)
Supplement: Supplementary file 3 [file DataSheet5.docx]

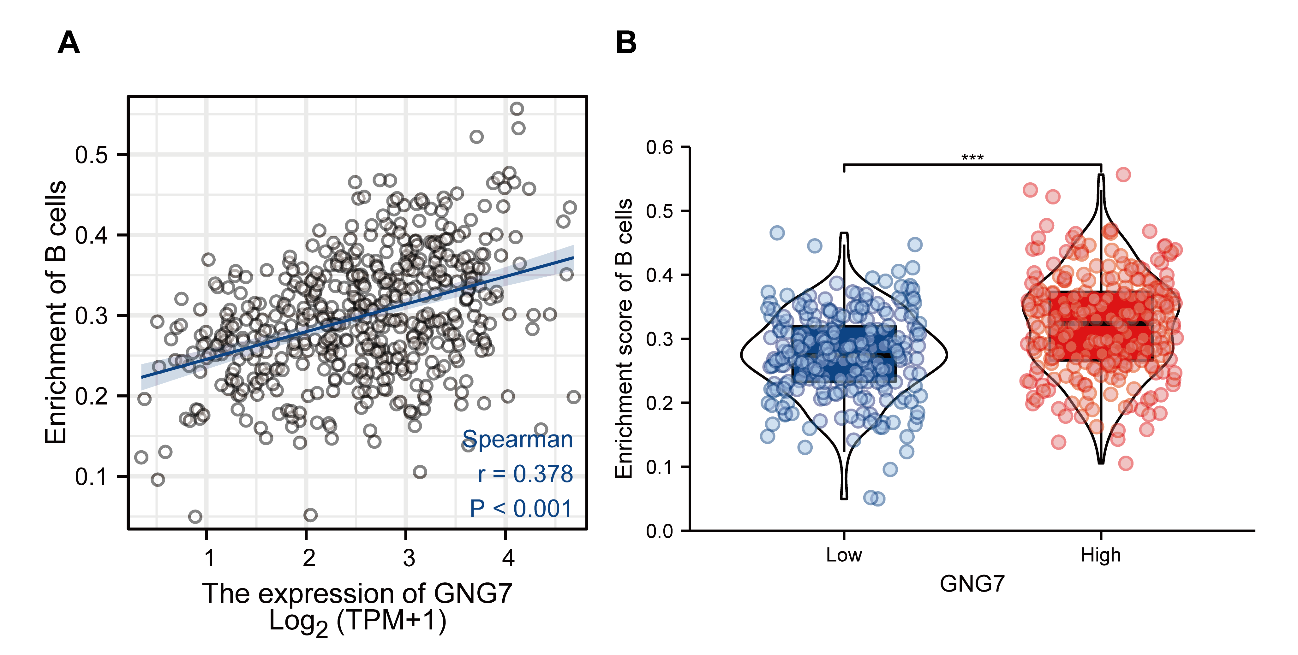


**Supplementary Figure 5. Correlation between the level of GNG7 expression and the level of B-cell immune infiltration in LUAD.** **(A)** Relationships between the infiltration level of B cell and GNG7 expression profiles in LUAD by Spearman’s analysis. **(B)** Comparison of B-cell infiltration levels between GNG7-high and GNG7-low groups.
