# Supplementary material for: Comprehensive analysis to identify GNG7 as a prognostic biomarker in lung adenocarcinoma correlating with immune infiltrates
Source: Front Genet. 2022 Sep 9;13:984575. doi: 10.3389/fgene.2022.984575 (PMC9500342; doi:10.3389/fgene.2022.984575)
Supplement: Supplementary file 4 [file DataSheet2.docx]

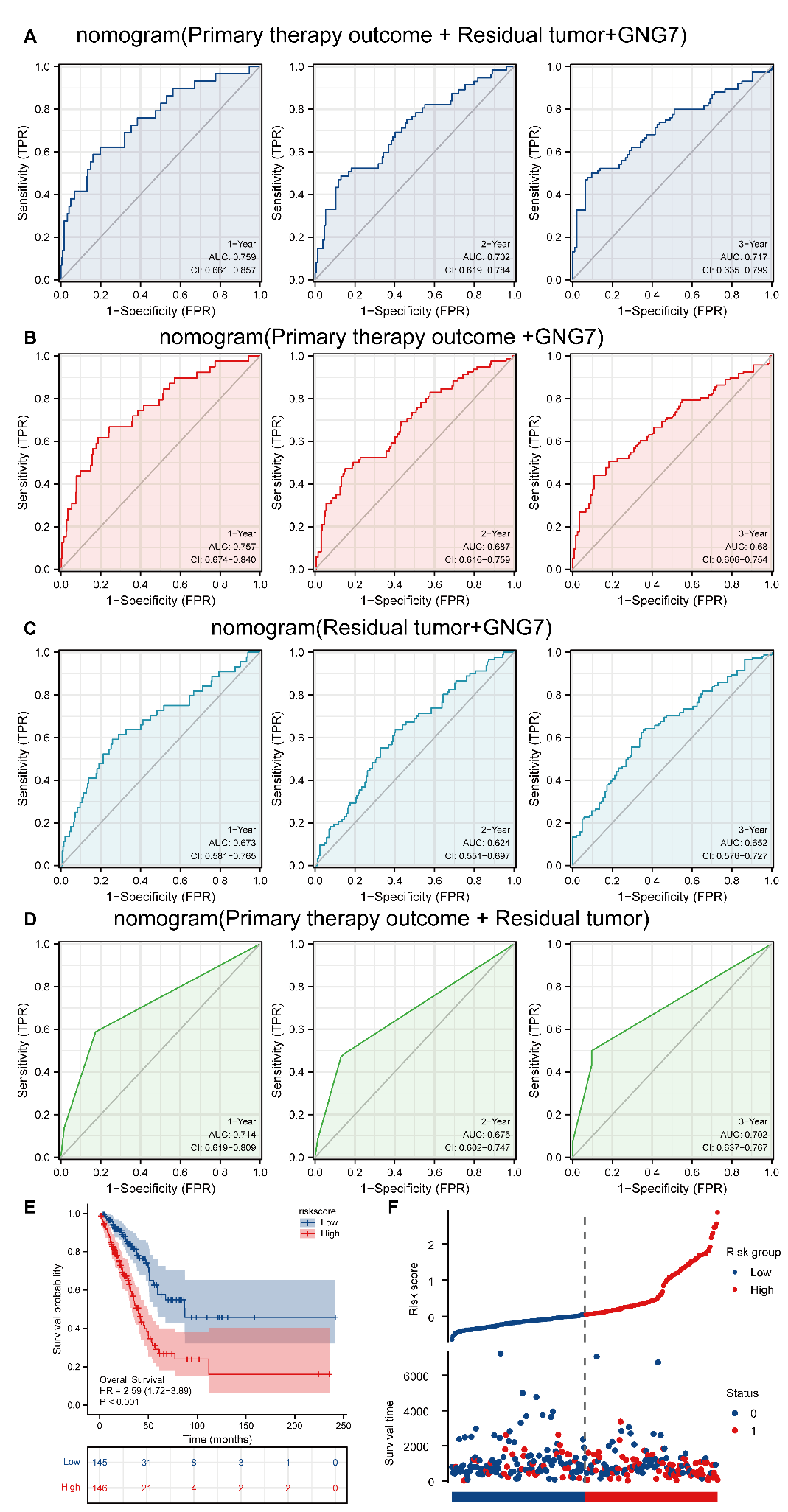


**Supplementary Figure 2 The comparative studies and validation of the clinical utility of the model (A)** The ROC curve of our constructed model consisting of Primary therapy outcome, Residual tumor and GNG7. **(B)** The ROC curve of the model consisting of Primary therapy outcome and GNG7. **(C)** The ROC curve of the model consisting of Primary therapy outcome and Residual tumor. **(D)** The ROC curve of the model consisting of Residual tumor and GNG7. **(E)** Survival curve of low- and high-risk groups stratified by median risk score. **(F)** The risk curve of low- and high-risk groups stratified by median risk score.
