# Supplementary material for: Comprehensive analysis to identify GNG7 as a prognostic biomarker in lung adenocarcinoma correlating with immune infiltrates
Source: Front Genet. 2022 Sep 9;13:984575. doi: 10.3389/fgene.2022.984575 (PMC9500342; doi:10.3389/fgene.2022.984575)
Supplement: Supplementary file 5 [file DataSheet1.docx]

**
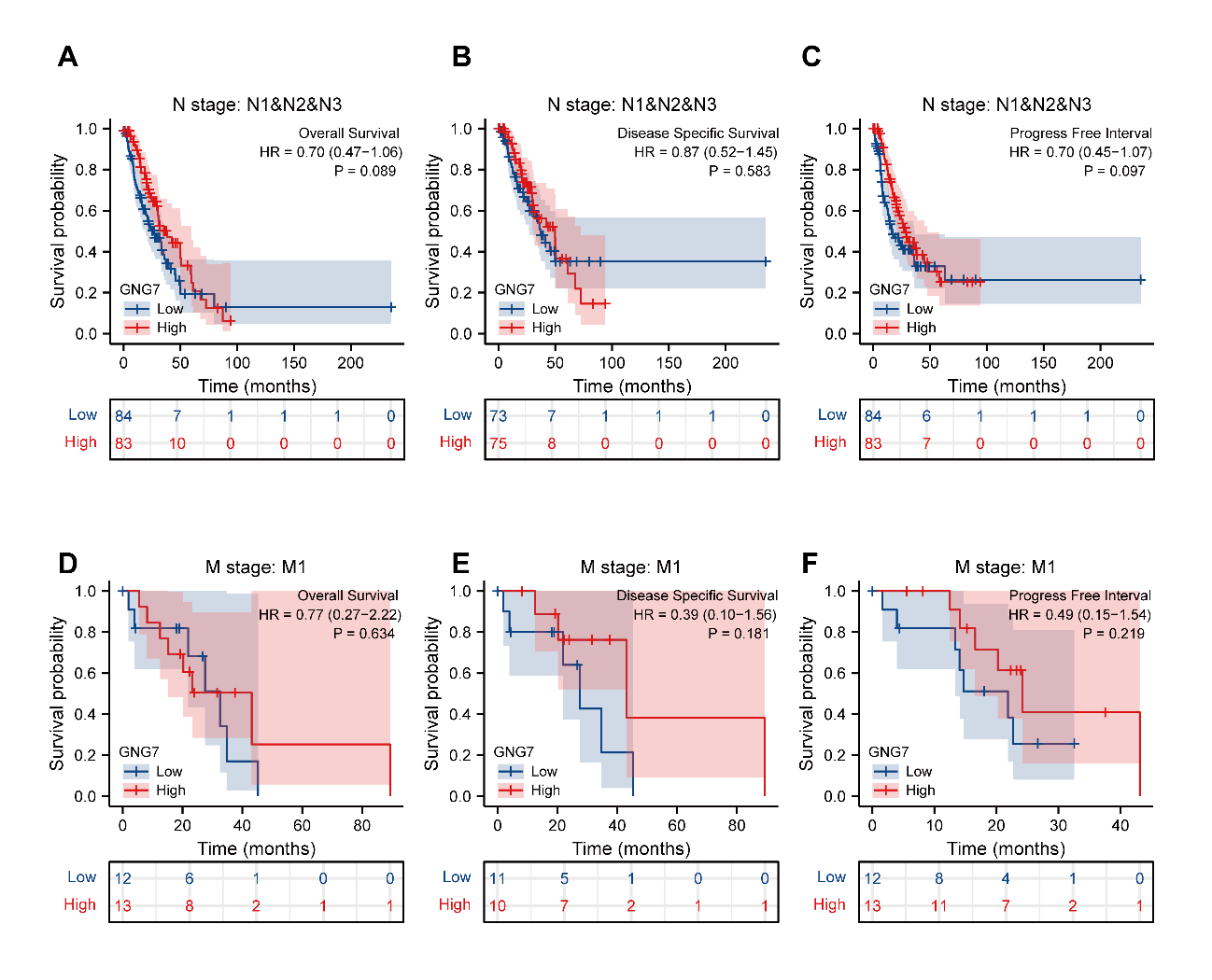
**

**Supplementary Figure 1. Subgroups analysis. (A-C)** The Kaplan-Meier curves of OS (A), DSS (B), and PFI (C) between GNG7-high and -low expression patients with LUAD in N1&N2&N3 stage. **(D-F)** The Kaplan-Meier curves of OS (D), DSS (E), and PFI (F) between GNG7-high and -low expression patients with LUAD in M1 stage.
